# Identification of novel arthropod vector G protein-coupled receptors

**DOI:** 10.1186/1756-3305-6-150

**Published:** 2013-05-24

**Authors:** Ronald J Nowling, Jenica L Abrudan, Douglas A Shoue, Badi’ Abdul-Wahid, Mariha Wadsworth, Gwen Stayback, Frank H Collins, Mary Ann McDowell, Jesús A Izaguirre

**Affiliations:** 1 Department of Computer Science & Engineering, University of Notre Dame, Notre Dame, IN, 46656, USA; 2 Department of Biological Sciences, University of Notre Dame, Notre Dame, IN, 46656, USA; 3 Eck Institute of Global Health, University of Notre Dame, Notre Dame, IN, 46656, USA

## Abstract

**Background:**

The control of vector-borne diseases, such as malaria, dengue fever, and typhus fever is often achieved with the use of insecticides. Unfortunately, insecticide resistance is becoming common among different vector species. There are currently no chemical alternatives to these insecticides because new human-safe classes of molecules have yet to be brought to the vector-control market. The identification of novel targets offer opportunities for rational design of new chemistries to control vector populations. One target family, G protein-coupled receptors (GPCRs), has remained relatively under explored in terms of insecticide development.

**Methods:**

A novel classifier, Ensemble*, for vector GPCRs was developed. Ensemble* was validated and compared to existing classifiers using a set of all known GPCRs from *Aedes aegypti*, *Anopheles gambiae*, *Apis Mellifera, Drosophila melanogaster*, *Homo sapiens*, *and Pediculus humanus*. Predictions for unidentified sequences from *Ae. aegypti*, *An. gambiae*, and *Pe. humanus* were validated. Quantitative RT-PCR expression analysis was performed on previously-known and newly discovered *Ae. aegypti* GPCR genes.

**Results:**

We present a new analysis of GPCRs in the genomes of *Ae, aegypti*, a vector of dengue fever, *An. gambiae*, a primary vector of *Plasmodium falciparum* that causes malaria, and *Pe. humanus*, a vector of epidemic typhus fever, using a novel GPCR classifier, Ensemble*, designed for insect vector species. We identified 30 additional putative GPCRs, 19 of which we validated. Expression of the newly discovered *Ae. aegypti* GPCR genes was confirmed via quantitative RT-PCR.

**Conclusion:**

A novel GPCR classifier for insect vectors, Ensemble*, was developed and GPCR predictions were validated. Ensemble* and the validation pipeline were applied to the genomes of three insect vectors (*Ae. aegypti*, *An. gambiae*, and *Pe. humanus*), resulting in the identification of 52 GPCRs not previously identified, of which 11 are predicted GPCRs, and 19 are predicted and confirmed GPCRs.

## Background

G protein-coupled receptors (GPCRs) are a class of seven transmembrane (7TM) proteins involved in signal transduction [1,2] that respond to a diverse assemblage of stimuli. These proteins play roles in essential invertebrate functions and are highly “drugable”, being targets for roughly 30% of drugs on the human pharmaceutical market [3]. The relative specificity of ligand binding combined with their abundance in metazoan genomes (1% of *Drosophila melanogaster* genome, 1.6% of *Anopheles gambiae* genome [4,5]) makes these proteins attractive targets for insecticide development. The availability of insect genomes enables the identification of novel targets such as GPCRs and rational drug design processes which can produce insecticides, repellents, and other products for the control of vectors such as *An. gambiae*[6,7].

We present a new genome-wide search for GPCRs in three important insect vectors responsible for the spread of diseases such as malaria (*An. gambiae*), dengue and yellow fever (*Aedes aegypti*) and typhoid fever (*Pediculus humanus*) [6,8,9]. Fredrikkson, *et al*. and Hill, *et al*. have identified GPCRs in the proteome of *An. gambiae*[5,10], Nene, *et al*. studied the GPCRs in *Ae. aegypti*[11], and Kirkness, *et al*. performed an initial analysis of the GPCRs in *Pe. humanus* as part of sequencing the genome [12]. Our analysis resulted in the identification of 52 additional GPCRs.

There are multiple *in silico* strategies for identifying potential GPCRs. Similarity based searches (e.g., BLAST) are limited in their ability to identify seven transmembrane (7TM) proteins, GPCRs included, due to the low degree of sequence conservation [1,13]. GPCRs have also been identified using short conserved sub-sequences, or motifs [13]. These GPCR “fingerprints” are defined by sets of motifs localized to transmembrane helices and intra and extracellular loops [14-16]. Fingerprints have been useful in identifying GPCRs and their associated classes and subfamilies. In addition, fingerprints can be used for screening out false positive GPCR predictions by requiring that an identified sequence contains all of the appropriate GPCR motifs. However, GPCR fingerprints have proven difficult to identify due to low sequence conservation as more GPCRs in each family are discovered and tend to be poor at identifying atypical or novel GPCRs with low homology to known GPCR family members.

Methods that rely on predicted sequence topology have proven more useful in the identification of GPCRs than those relying on primary sequence alone. Classifiers such as HMMTOP [17] and TMHMM [18] predict transmembrane helices and intracellular and extracellular loops using Hidden Markov Models (HMM) and filter sequences based on the number of predicted transmembrane helices to identify potential 7TM proteins. Phobius [19,20] offers more functionality than either HMMTOP or TMHMM by including the identification of signal peptides for use in screening out false positive predictions. Signal peptides are composed of a hydrophobic region flanked by hydrophilic regions followed by a cleavage site motif and are often incorrectly categorized as membrane spanning regions when not taken explicitly into account [18-21]. Although these 7TM protein classifiers have been used to identify GPCRs, they are not able to distinguish between GPCRs and other types of 7TM proteins, such as ion channels, aquaporins, and ATPases [22].

GPCRHMM uses an HMM specific to GPCRs [21]. In addition to predicting the topology and number of transmembrane helices, GPCRHMM uses the predicted loop lengths (it assumes a median of 22-24 amino acids per loop) and amino acid composition as additional filters. GPCRHMM produces two numbers, a global score and a local score, and a Boolean

prediction based on default cutoffs for each score. Whereas the global score is based on the HMM match of the entire protein, the local score excludes the signal peptide and N- and C-termini models and is used to improve discrimination between GPCRs and false positives such as cysteine-rich proteins. By utilizing these characteristics specific to GPCRs to distinguish between GPCRs and other 7TM proteins, GPCRHMM is able to more accurately classify input sequences than HMMTOP, TMHMM, and Phobius.

PredCouple was originally designed to predict the family of G-proteins with which a given GPCR will bind [23,24]. PredCouple utilizes a preliminary step based on HMMs from the Pfam database [25,26] to screen out non-GPCRs, a filtering capability on par with other methods such as GPCRHMM, thus making PredCouple useful as a GPCR classifier.

Several “alignment-free” methods exist that do not depend on comparing the primary sequence or the topology to known GPCRs [27]. One such example is the Quasi-periodic Feature Classifier (QFC) that utilizes a sliding window approach to scan the entire proteome and identify membrane-associated proteins based on quasi-periodic physiochemical properties of amino acids [28]. Lapinsh and colleagues also developed an alignment-free method that utilizes physiochemical properties of proteins [29].

The performance of individual classifiers has been improved by combining multiple classifiers into a pipeline or ensemble. The whole-proteome and subset GPCR repertoires of multiple organisms including *Homo sapiens* (human), *Mus musculus* (mouse), *Danio rerio* (zebra fish), *Ratus norvegicus* (rat), *Canis familiaris* (dog), *Gallus gallus* (chicken), and *Tetraodon nigroviridis* (puffer fish) have been identified or extended using a combination of BLAST with known GPCRs (often from *Ho. sapiens* or *Dr. melanogaster*) or HMMs trained from known GPCRs or from the Pfam database [10,30-40]. Inoue, *et al.* demonstrated that the combination of the HMMTOP and TMHMM 7TM classifiers can be used to more accurately distinguish between GPCRs and the larger class of 7TM proteins than either classifier individually [22]. Moriyama, *et al.* identified 394 7TM proteins in the *Arabidopsis thaliana* proteome by combining multiple 7TM classification methods, including alignment-based and alignment-free methods [41]. Gookin, *et al*. developed and applied a pipeline utilizing the classifiers QFC, HMMTOP, Phobius, TMHMM, and GPCRHMM to perform a proteome-wide computational analysis of GPCRs in *Ar. thaliana*, *Oryza sativa*, and *Populus trichocarpa*[42]. Previous studies have identified GPCRs in the *An. gambiae* proteome using QFC [5] and a combination of BLAST against known GPCRS and HMMs derived from GPCRs [10]. GPCRs in the *Ae. aegypti* proteome have been identified with a combination of QFC and tBLASTn queries against known GPCRs from *An. gambiae*, *Dr. melanogaster*, and *Bombyx mori*[11]. In the *Pe. humanus* proteome, GPCRs were identified using tBLASTn queries against known GPCRs from *Ae. aegypti*, *An. gambiae*, and *Dr. melanogaster*[12].

We began by evaluating existing GPCR classifiers such as GPCRHMM [21] and PredCouple [23,24]. The sensitivity and accuracy of these classifiers was reduced for vector species, which was expected considering they were not trained on these organisms. We developed a novel ensemble classifier, Ensemble*, for insect vector GPCRs that combines and improves upon the prediction capabilities of GPCRHMM and the Pfam A GPCR Clan Hidden Markov Models (HMMs) [26]. When evaluated against GPCRHMM and PredCouple, Ensemble* demonstrated higher sensitivity and accuracy. Putative GPCRs were identified in the vector predicted proteomes using Ensemble*, while a novel pipeline was used to validate and confirm the predictions. Expression of the newly discovered *Ae. aegypti* GPCR genes was confirmed in head and body tissues via quantitative RT-PCR.

These results will be of interest to the research community due to their potential applicability to insect vector population control via insecticide development [43]. Furthermore, Ensemble* identified a high number of previously unidentified GPCRs in vector species. The availability of better tools for the identification of signal transduction proteins such as GPCRs will be valuable as more insect genomes are sequenced.

## Results and discussion

**Figure 1:**
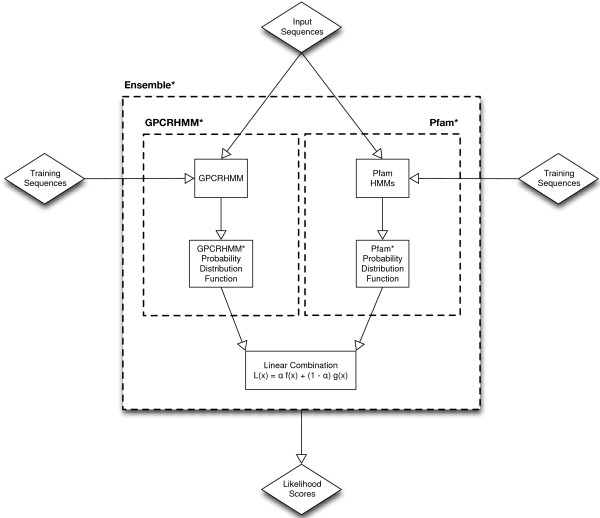
Flowchart describing the Ensemble*, GPCRHMM*, and Pfam* classifiers. The diamonds represent sequences, while the rectangles represent steps of the classifiers. The dashed rectangles describe to which classifiers the components belong. The input sequences are the sequences to be classified, while the training sequences are sequences which have known designations of either GPCR or not GPCR.

Multiple classification methods exist for identifying GPCRs. Two particular classification methods, GPCRHMM and the Pfam A GPCR clan hidden Markov models (HMMs), are both accurate and sensitive on their own but do not necessarily predict the same set of sequences as GPCRs. We developed a new classifier, Ensemble*, that combines the prediction capabilities of GPCRHMM and the Pfam A GPCR clan Hidden Markov Models (HMMs) to improve the accuracy and sensitivity of predicting GPCRs from *in silico* peptide translations of vector genomes. Discrete likelihood score functions were developed incorporating likelihood scores from the GPCRHMM global scores and logarithms of Pfam HMM e-values. The discrete likelihood scores were combined using a linear weighting to produce an overall likelihood score (Figure 1).

### Formation of test sets

For training and validation of the classifiers, we assembled test sets that contained validated GPCR sequences (Table 1). *Ae. aegypti*, *An. gambiae*, and *Pe. humanus* were chosen as representative vector organisms. Published GPCR sequences from *An. gambiae*[5,44], *Ae. aegypti*[11], and *Pe. humanus*[12], VectorBase [45,46] searches for GPCR annotations and GO terms, and GPCRDB [47,48] searches were used as sources for compiling the test sets for these organisms.

**Table 1:** Data set sources and construction

**Organism**	**Predicted proteome source and version**	**Training set source**
*Ae. aegypti*	VectorBase, v. AaegL1.2	VB and GPCRDB annotations
*An. gambiae*	VectorBase, v. AgamP3.5	[5], VB and GPCRDB annotations
*Ap. mellifera*	Beebase, v. Pre-Release 2 OGS	GPCRDB annotations
*Dr. melanogaster*	Flybase, v. 5.29	Flybase and GPCRDB annotations
*Ho. sapiens*	Ensembl, v. 37.59	[49], Ensembl and GPCRDB annotations
*Pe. humanus*	VectorBase, v. 1.2	VB and GPCRDB annotations

For further validation, the GPCR datasets of the honey bee, *Ap. mellifera*, the fruit fly, *Dr. melanogaster*, and humans were chosen because they are well annotated and used as the basis of other GPCR studies [10,30-40]. GPCRDB was used to find GPCRs from *Ap. mellifera* and *Dr. melanogaster*. Additional GPCRs from *Dr. melanogaster* were found by searching Flybase [4,50-53] for GO terms. The positive test set for *Ho. sapiens* consisted of GPCRs identified through a search of Ensembl [54] for GO terms and sequences identified by Zhang *et al*. [49].

### Design of the ensemble* classifier

After evaluating the existing classifiers, we designed a new classifier, Ensemble*, that improves prediction sensitivity and accuracy on vector organisms. An ensemble approach which combines multi-classifiers was chosen. Several of the best-performing existing classifiers use different methods for predicting GPCRs which results in recognizing different but overlapping sets of GPCRs. The identification of the same sequence by multiple classifiers provides more confidence (increases accuracy) in the prediction, while the ability of any classifier to identify a sequence not found by the other increases the number of predictions (sensitivity). We required that the Ensemble* classifier provides a discrete likelihood score between 0 and 1 for each sequence, indicating the confidence level of the prediction. Thus, we chose classifiers for the ensemble that provided discrete scores that could be used to determine prediction confidence.

We determined that the two best pre-existing classifiers were GPCRHMM and PredCouple.

GPCRHMM outputs a “raw” global score that fits our requirements for a meaningful discrete confidence score. We designed GPCRHMM* as an intermediate classifier that maps GPCRHMM’s global score to a likelihood score between 0 and 1 based on the known status of proteins in the combined training set. Unlike GPCRHMM, PredCouple only provides a boolean prediction indicator and not a discrete score [23,24]. As PredCouple uses Pfam Hidden Markov Models (HMMs) for seven transmembrane and GPCR proteins, we utilized the Pfam HMMs as the second classifier in the ensemble. HMMER [55] is used to match input sequences against the Pfam HMMs and provides an expectation value (e-value) for each sequence giving the probability that a sequence would match the HMM if the sequence had been generated randomly. A lower e-value indicates a better match. We used the default e-value given by HMMER [55] as the threshold; sequences with e-values above the threshold were not considered to be GPCRs. In a manner similar to GPCRHMM*, we developed Pfam* as an intermediate classifier that maps the logarithm of the e-values to likelihood scores.

Ensemble* was developed by combining GPCRHMM* and Pfam* (Figure 1). A simple linear-weighting was used: Ensemble*’s likelihood scores are computed by multiplying and then adding the likelihood scores of GPCRHMM* and Pfam* by the weights 1 - α (GPCRHMM*) and α (Pfam*), respectively.

The Ensemble* classifier offers several features that make it advantageous when compared with the other classifiers. First, the confidence of each prediction for each sequence is represented as a discrete likelihood score normalized to a value between 0 and 1. Inexperienced users can easily interpret the discrete likelihood score, while experienced users can use the information provided by the discrete likelihood score to provide more advanced analysis. For example, we found it was useful to identify a threshold value such that only sequences with predicted likelihood scores higher than or equal to that threshold value were classified as predicted GPCRs. However, a user may choose to sort predictions into more nuanced categories such as high, neutral, and low confidence predictions. The discrete likelihood score also allows the Ensemble* classifier to be more easily incorporated into a pipeline or other analysis tools. Secondly, Ensemble* can be “tuned” for different needs through the choice of training set and the relative weights given to different component classifiers, i.e., GPCRHMM* and Pfam*. See the Additional file 1 for discussion concerning the choice of test sets and value, which determine the relative weights of GPCRHMM* and Pfam*.

### Ensemble* classifier improves prediction performance on combined dataset

**Figure 2:**
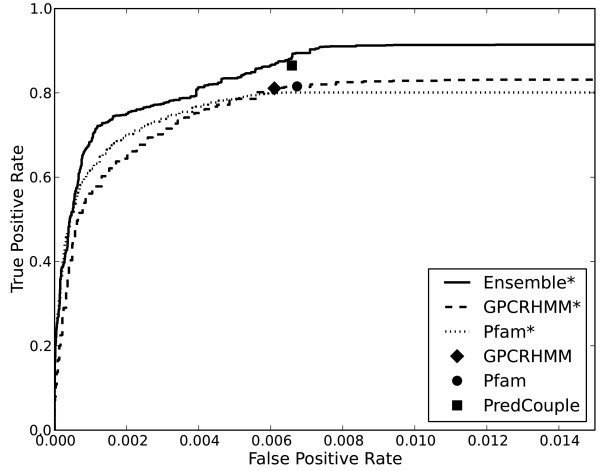
**ROC Curve comparing classifier performance on combined data set.** The dashed (GPCRHMM*), dotted (Pfam*), and solid (Ensemble*) curves represent the relationship between the percentage of training set GPCRs correctly predicted (true positives) plotted against the percentage of sequences miss-classified as GPCRs (false positives) by the classifiers as the likelihood score threshold, was decreased from 1 (accepting nothing) to 0 (accepting everything). GPCRHMM (diamond), Pfam (circle), and PredCouple (square) only produce true/false predictions for each sequence and hence are represented as single points.

Ensemble*’s overall classification performance was evaluated on the combined inferred proteomes of the organisms *Ae. aegypti*, *An. gambiae*, *Ap. mellifera*, *Dr. melanogaster*, *Ho. sapiens*, and *Pe. humanus*. GPCRHMM*, Pfam*, and Ensemble* were trained using the combined data set, while GPCRHMM, Pfam, and PredCouple were used as provided by their authors. By training and evaluating the classifiers on the same data set, we are able to test the ability of the classifiers to exactly reproduce the classifications of the sequences. The Ensemble* classifier achieved a higher true positive rate than the other classifiers for the same false positive rates (Figure 2). For this evaluation, we considered a sequence predicted as a GPCR by a classifier but not in the test set to be a “false positive,” while a “true positive” is when a sequence in the test set is correctly predicted to be a GPCR.

To enable direct comparison of the classifiers, we evaluated each classifier at the false positive rates generated by GPCHMM (0.0061), Pfam (0.0067), and PredCouple (0.0064) on the combined training set, and 0.01, the false positive rate at which Ensemble*’s true positive rate plateaus (Table 2). Ensemble* had a true positive rate (87%) at least 6% higher than GPCRHMM (81%), GPCRHMM* (81%) and Pfam* (80%) at GPCRHMM’s false positive rate (0.0061). Using Pfam’s false positive rate (0.0067), Ensemble* had a true positive rate (89%) at least 7% higher than GPCRHMM* (81%), Pfam (82%), and Pfam* (80%). When compared at PredCouple’s false positive rate (0.0064), Ensemble* had a true positive rate (89%) at least 3% higher than GPCRHMM* (81%), Pfam* (80%), and PredCouple (86%). At 0.01, where Ensemble*’s true positive rate plateaus at 91%, Ensemble* performed at least 11% better than GPCRHMM* at 83% and Pfam* at 80%. When comparing the highest true positive rates found by each of the classifiers, Ensemble* (91%) had a true positive rate at least 5% higher than that of GPCRHMM (81%), GPCRHMM* (81%), Pfam (82%), Pfam* (80%), and PredCouple (86%). Overall, Ensemble* correctly identified more GPCRs and with greater accuracy than any of the other classifiers.

**Table 2:** Classifiers’ true positive rates for different false positive rates

**Classifier**	True Positive Rate (TPR)*				
	**GPCRHMM’s FPR (0.0061)**	**PredCouple’s FPR (0.0066)**	**Pfam’s FPR (0.0067)**	FPR where Ensemble*’s TPR plateaus (0.01)	**Best for all FPRs ≤ 0.01**
Ensemble*	87%	89%	89%	91%	91%
GPCRHMM	81%	--	--	--	81%
GPCRHMM*	81%	81%	81%	83%	83%
Pfam	--	--	82%	--	82%
Pfam*	80%	80%	80%	80%	80%
PredCouple	--	86%	--	--	86%

*Percentage of training set GPCRs correctly predicted (true positive rates) for fixed percentages of sequences misclassified as GPCRs (false positive rates) are given. The false positive rates (FPR) of GPCRHMM, PredCouple, Pfam, and at which Ensemble*’s true positive rate plateaus were chosen to provide the most direct comparisons between the classifiers.

### Prediction performance improvement is more marked for vector sequences

**Figure 3:**
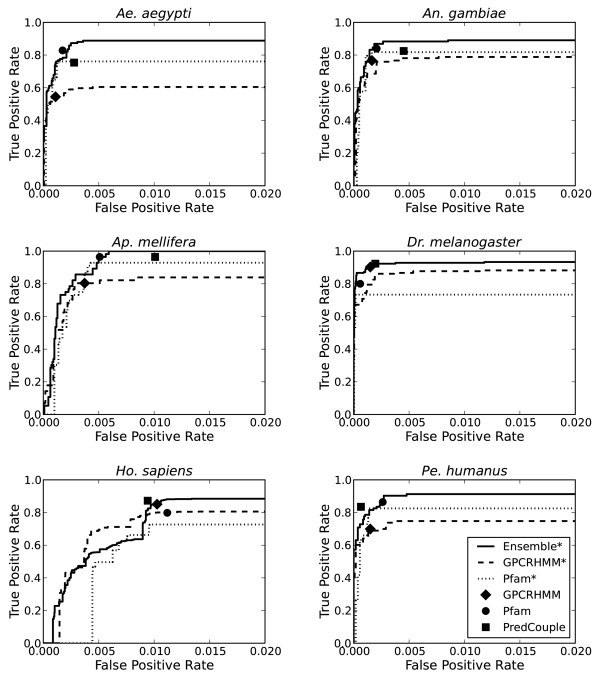
**ROC Curves comparing classifier performance on individual organisms.** For each ROC curve, the classifiers were trained on five of the organisms and then evaluated on the sixth organism (given in the title). The solid (Ensemble*), dashed (GPCRHMM*), and dotted (Pfam*) lines represent the relationship between the percentage of correctly predicted training set GPCRs (true positives) and the percentage of incorrectly predicted non-GPCRS (false positives) as the likelihood score threshold is decreased from 1 (accept nothing) to 0 (accept everything). GPCRHMM (diamond), Pfam (circle), and PredCouple (square) only produce true/false predictions for each sequence and hence are represented as single points.

Efficacy was evaluated in two ways. First, a per organism hold-out validation was performed to simulate realistic use cases where the classifiers will be used to identify GPCRs in the predicted proteomes of novel organisms after being trained on proteomes of organisms for which the GPCRs are known. Six validation trials were performed where sequences from one species was designated as the validation holdout set, while sequences from the remaining five organisms were used to train the classifiers. Ensemble* showed marked improvement in sensitivity of at least 13%, 6%, 7%, and 8%, respectively, for *Ae. aegypti* (89%), *An. gambiae* (89%), and *Pe. humanus* (91%) GPCRs when compared with GPCRHMM (54%, 77%, 70%), Pfam (83%, 84%, 86%), PredCouple (75%, 82%, 83%), Pfam* (76%, 82%, 83%), and GPCRHMM* (60%, 79%, 75%) (Figure 3).

It is particularly interesting to note that GPCRHMM performed particularly poorly on the *Ae. aegypti* (54%) and *Pe. humanus* (70%) sequences - Ensemble* identified at least 35% more of the test set sequences for *Ae. aegypti* and 21% more for *Pe. humanus*. As GPCRHMM uses a number of specific features (e.g., length, amino acid composition, sequence similarity in transmembrane regions) and was trained on sequences from *Dr. melanogaster* and *Ho. sapiens*, it is likely that the internal model used by GPCRHMM is too specific to the features of the GPCRs of those organisms that GPCRHMM is excluding valid sequences from other organisms. In contrast, the Pfam HMMs depend on sequence similarity which rewards similarity rather than penalizing differences. By combining the two approaches, Ensemble* is able to take advantage of their strengths while overcoming their disadvantages to identify more GPCRs than any other classifier individually.

Secondly, we compared the number of test set sequences that Ensemble*, GPCRHMM, Pfam, and PredCouple failed to predict as GPCRs (“false negatives”) by species (Table 3). GPCRHMM, Pfam, and Predcouple were run with the default settings, while a positive likelihood score was considered a prediction for Ensemble*. Ensemble* missed fewer sequences than all of the other classifiers for all six species and was able to find all of the *Ap. mellifera* test set sequences.

**Table 3:** Number of test set sequences found / missed by species

**Species (Total sequences)**	Number of sequences found / Missed*			
	**GPCRHMM**	**Pfam**	**Predcouple**	Ensemble*
*Ae. aegypti* (134)	73 / 61	111 / 23	101 / 33	**122 / 12**
*An. gambiae* (137)	105 / 32	115 / 22	113 / 24	**122 / 15**
*Ap. mellifera* (56)	45 / 11	54 / 2	54 / 2	**56 / 0**
*Dr. melanogaster* (195)	176 / 19	156 / 39	180 / 15	**185 / 10**
*Ho. sapiens* (892)	759 / 133	712 / 180	778 / 114	**807 / 85**
*Pe. humanus* (103)	72 / 31	89 / 14	86 / 17	**95 / 8**
Vectors (374)	250 / 124	315 / 39	300 / 74	**339 / 35**
Total (1517)	1230 / 287	1237 / 280	1312 / 205	**1387 / 130**

* The number of test set sequences each classifier identified (found) and was unable to identify (missed) as GPCRs are given by species, vectors, and total. GPCRHMM, Pfam, and Predcouple were run using default settings. For Ensemble*, sequences with a positive likelihood score were considered to predicted GPCRs. The best results are in bold.

### Independent validation confirms prediction of putative GPCRs

**Figure 4:**
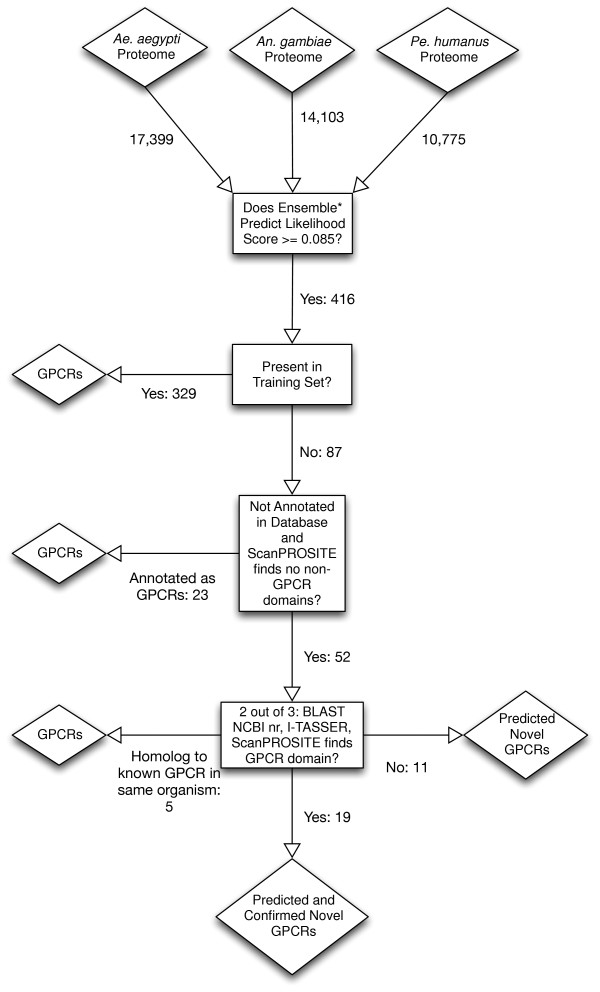
**GPCR Validation pipeline.** Diamonds indicate input and output sequences. Rectangles represent the validation programs and filters. Arrows indicate the movement of sequences and are labeled with the number of sequences that passed from one validation step to the next. Numbers for sequences that were identified as something other than GPCRs are not shown. The first filter identifies sequences which are annotated as GPCRs in the database and removes any sequences which were annotated as something other than GPCRs or were identified by ScanPROSITE as having non-GPCR domains. The second filter uses a combination of the BLAST hits, I-TASSER results, and identification of GPCR domains by ScanPROSITE to categorize the final 52 predicted but unconfirmed sequences.

A multi-step validation process combining database annotations, similarity searches, domain identification, and structure prediction of the Ensemble* classifier predictions was performed (Figure 4). A total of 1,369 arthropod sequences, of which 697 belong to the vector species, had positive likelihood scores. Using a threshold value of 0.085, 416 (148 for *Ae. aegypti*; 148 for *An. gambiae*; and 120 for *Pe. humanus*) sequences were predicted as putative vector GPCRs. Of the 416 predicted sequences, 329 were in the original training set, confirming them as known GPCRs, (Figure 4). Eighty-seven predicted sequences were not in the training set and required further validation and confirmation. Of these 87 predicted sequences, 12 sequences were false positives: either their database annotation or identification of domains by ScanPROSITE [56] indicated the sequences as something other than a GPCR. From their respective database annotations, 23 predicted sequences were identified as previously-known GPCRs. Of the remaining 52 sequences, 27 were validated as having GPCR domains by ScanPROSITE but did not have a database GPCR annotation and 25 could be neither confirmed by their respective database annotation nor validated by ScanPROSITE as containing a GPCR domain (Additional file 1: Table S1).

**Figure 5:**
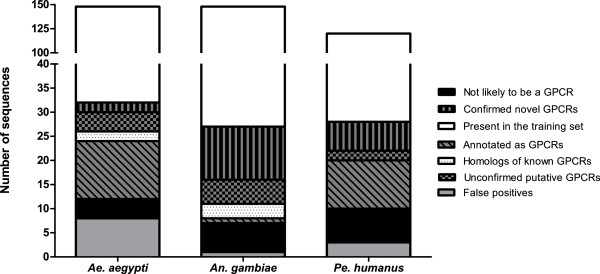
Distribution of the newly-discovered vector GPCR sequences according to their GPCR status for each vector species.

The 52 predicted but unconfirmed sequences were validated using a combination of a BLAST search of the NCBI nr database, ScanPROSITE, and I-TASSER [57]. Due to the high divergence among GPCR protein sequences, I-TASSER was employed to identify putative protein structure similarity of the 52 predicted sequences to known GPCR protein structures. Five of the sequences were homologs to known GPCRs from the same organisms and are therefore likely to be duplicate sequences. Seventeen sequences are not likely to be GPCRs as their closest homolog was either confirmed as something explicitly other than a GPCR or sequences which were not likely to be GPCRs. Thirty predicted GPCR sequences that have no homologs within the same organism were considered to be newly-discovered (previously-unidentified) vector GPCRs (Table 4). Nineteen of the 30 predicted GPCRs have been confirmed as putative GPCRs by at least two of the *in silico* methods. The independent validation results for the 416 predicted sequences have been summarized in Figure 5. The previous studies may have missed these GPCRs due to use an older version of the official gene set.

**Table 4:** Newly-discovered GPCRs identified by ensemble*†

**Sequence ID**	**Species**	**Prediction likelihood value**	**Evidence**	**BLAST sequence name**
AAEL013430-PA	*Ae. aegypti*	0.346	ScanPROSITE and BLAST and I-TASSER	putative GPCR class a orphan receptor 5
AAEL000818-PA	*Ae. aegypti*	0.188	ScanPROSITE and BLAST	class C metabotropic glutamate-like G- protein coupled receptor GPRmgl4, putative
AGAP002229-PA	*An. gambiae*	0.691	ScanPROSITE and BLAST	serotonin receptor
AGAP004930-PA	*An. gambiae*	0.658	ScanPROSITE and BLAST and I-TASSER	G protein coupled receptor
AGAP000383-PA	*An. gambiae*	0.480	ScanPROSITE and BLAST	G protein coupled receptor
AGAP005229-PA	*An. gambiae*	0.440	ScanPROSITE and BLAST and I-TASSER	G protein coupled receptor
AGAP000606-PA	*An. gambiae*	0.411	ScanPROSITE and BLAST	alpha-2 adrenergic receptor
AGAP013324-PA	*An. gambiae*	0.409	ScanPROSITE and BLAST	G protein-coupled receptor
AGAP008703-PA	*An. gambiae*	0.278	ScanPROSITE and BLAST and I-TASSER	G protein-coupled receptor
AGAP008237-PA	*An. gambiae*	0.250	BLAST and I- TASSER	G protein-coupled receptor 143
AGAP011320-PA	*An. gambiae*	0.236	ScanPROSITE and BLAST and I-TASSER	Beta-3 adrenergic receptor
AGAP012824-PA	*An. gambiae*	0.167	ScanPROSITEand BLAST and I-TASSER	tachykinin receptor
AGAP011646-PA	*An. gambiae*	0.112	ScanPROSITE and BLAST	class C metabotropic glutamate-like G-protein coupled receptor
PHUM074100-PA	*Pe. humanus*	0.375	ScanPROSITE and BLAST	Frizzled-7
PHUM010590-PA	*Pe. humanus*	0.342	ScanPROSITE and I-TASSER	PREDICTED: cadherin EGF LAG seven-pass G-type receptor 3-like
PHUM423330-PA	*Pe. humanus*	0.289	ScanPROSITE and I-TASSER	N/A
PHUM447490-PA	*Pe. humanus*	0.250	ScanPROSITE and I-TASSER	neuropeptide receptor A31
PHUM618870-PA	*Pe. humanus*	0.208	ScanPROSITE and I-TASSER	G protein-coupled receptor
PHUM128700-PA	*Pe. humanus*	0.167	ScanPROSITE and I-TASSER	G protein-coupled receptor
AAEL005994-PA	*Ae. aegypti*	0.278	Unconfirmed	uridine cytidine kinase i
AAEL002694-PA	*Ae. aegypti*	0.250	Unconfirmed	transmembrane protein 87A
AAEL000851-PA	*Ae. aegypti*	0.217	Unconfirmed	N/A
AAEL010852-PA	*Ae. aegypti*	0.214	Unconfirmed	Transmembrane protein 145
AGAP000130-PA	*An. gambiae*	0.430	Unconfirmed	PREDICTED: latrophilin 2-like
AGAP005356-PA	*An. gambiae*	0.423	Unconfirmed	PREDICTED: alpha-1A adrenergic receptor-like
AGAP011701-PA	*An. gambiae*	0.400	Unconfirmed	GPRmgl4
AGAP004783-PA	*An. gambiae*	0.227	Unconfirmed	neuropeptide receptor A16
AGAP007896-PA	*An. gambiae*	0.141	Unconfirmed	sprinter
PHUM596180-PA	*Pe. humanus*	0.476	Unconfirmed	transmembrane protein 145
PHUM288590-PA	*Pe. humanus*	0.136	Unconfirmed	N/A

† Sequences were predicted using the Ensemble* classifier. Independent validation was performed using ScanPROSITE, I-TASSER, and homology to GPCRs in other organisms as determined by a BLAST search of the NCBI nr database. Status as a newly-discovered GPCR was contingent upon identification by the Ensemble* classifier and the existence of no contrary evidence, while confirmed GPCRs also had to be validated by at least two independent methods.

Forty-seven, or 11%, of the vector training set sequences were not identified as GPCRs as they did not have likelihood scores above the threshold. There are several possible reasons for the omission of the 47 sequences, including sequences that were too short to accurately determine if they were GPCRs, too much divergence from known GPCR sequences and structural features, and positive likelihood scores less than the threshold value.

### Thirty previously-unidentified vector GPCRs were predicted by the classifier

Ensemble* predicted 30 previously-unidentified putative vector GPCRs of which 19 were confirmed (2 for *Ae. aegypti*, 11 for *An. gambiae*, and 6 for *Pe. humanus*). While GPCRs from *Ae. aegypti* and *An. gambiae* sequences were used as part of the training sets for the Pfam HMMs, *Pe. humanus* sequences were not. Most of the *Pe. humanus* predicted GPCR sequences (Table 4) were confirmed as GPCRs by using motif identification (ScanPROSITE) and protein modeling (I-TASSER); only one of the six *Pe. humanus* GPCR predictions lacked a good match in the NCBI nr database. The identification of *Pe. humanus* putative GPCRs that had no close homologs in the NCBI nr database indicated the improved ability of Ensemble* to identify GPCRs in a novel organism. The usefulness of Ensemble* to identify new GPCRs in already well-studied organisms was demonstrated by the prediction of 13 new GPCRs in the two mosquito species: *Ae. aegypti* and *An. gambiae*. The remaining 11 (4 for *Ae. aegypti*, 5 for *An.*

*gambiae*, and 2 for *Pe. humanus*) putative GPCRs could not be confirmed due to low levels of similarity to known GPCR structures or sequences. The identification of additional GPCRs by Ensemble* is likely due to the improved sensitivity in comparison with other classifiers and the use of newer gene sets with improved gene annotations. As the gene sets for *Ae. aegypti*, *An. gambiae*, and *Pe. humanus* improve, Ensemble* may be able to identify more GPCRs.

### Predicted GPCR genes are expressed

Expressed sequence tags (ESTs) represent fragments of cDNA that were generated by reverse transcribing mRNA available in the cells or organisms being analyzed. A match against an EST sequence in a database indicates the sequence is expressed and the organism at some point is likely synthesizing the equivalent protein in its lifetime. In the current study, matches against the VectorBase respective EST datasets were found for only two of the three vector species of interest: *An. gambiae* and *Ae. aegypti*, respectively. One hundred and forty six (146, 35%) of the 416 vector GPCRs predicted by the Ensemble* classifier had EST matches in VectorBase. No EST matches were found for *Pe. humanus*, likely due to the smaller number of EST studies that have been performed on the species.

Sixty-three *Ae. aegypti* sequences were validated as GPCRs but had no EST matches in VectorBase, including 10 sequences that were not part of the original training set. These ten, plus an additional 28 randomly-selected sequences were assessed by quantitative RT-PCR. All but one of these 38 selected sequences were expressed in either the mosquito head, body, or both (Additional file 1: Table S2).

## Conclusions

Ensemble*, a novel GPCR classifier for insect vectors, was developed. A validation pipeline was described and used to validate the predictions of Ensemble*. As the genomes of more vector species are sequenced, the availability of better tools for predicting and validating GPCRs such as the Ensemble* classifier and the validation pipepline presented here will continue to be of great interest.

We also provided a new analysis of the GPCR repertoires of the three vector species, *Ae. aegypti*, *An. gambiae*, and *Pe. Humanus,* which resulted in the discovery of 30 new vector GPCRs. Annotations for newly-discovered GPCRs were submitted to VectorBase. EST expression analysis were used to demonstrate that the sequences predicted by Ensemble* and validated by the pipeline corresponded with expressed genes. Given the importance of arthropod vectors to human health, we believe the identification of these additional vector GPCRs should be useful to the research community.

## Methods

### Data sets

Positive training sets of known GPCRs were built from multiple sources, including published GPCR sequences from *An. gambiae*[5,44], *Ae. aegypti*[11], and *Pe. humanus*[12], searching VectorBase [45,46] for GPCR annotations and GO terms G-protein coupled receptor activity (GO:0004930), G-protein coupled receptor signaling pathway (GO:0007186), and receptor activity (GO:0004872), and querying GPCRDB [47,48]. Duplicate entries were identified and removed using BLAST. For *Ap. mellifera*, we obtained the pre-release 2 *in silico* peptide translations of the genome from Beebase [58,59]. A positive training set of known GPCRs was then compiled from a search of GPCRDB. A positive training set of GPCRs for *Dr. melanogaster* was compiled by searching GPCRDB and Flybase [4,50-53] for sequences annotated with the above GO terms. The *in silico* peptide translations of the *Ho. sapiens* genome were obtained from Ensembl [54]. The positive training set for *Ho. sapiens* consisted of GPCRs identified through a search of Ensembl for GO terms (see above) and sequences identified by Zhang *et al*. [49].

The *in silico* peptide translations of the genomes for all of the organisms were current as of August 2010. For the purposes of this study, we assumed that odorant receptors were not GPCRs [60-62] and removed them from the positive training sets. The negative training sets for each organism were defined as the remaining sequences in the peptide translations from each organism.

### Development of the ensemble* classifier

Ensemble* combined the prediction capabilities of GPCRHMM and the Pfam A GPCR clan Hidden Markov Models (HMMs). Discrete likelihood score functions were used to map the GPCRHMM global scores and logarithms of the Pfam HMM e-values to likelihood scores. The discrete likelihood scores were combined using a linear weighting to produce an overall likelihood score.

#### GPCRHMM*

GPCRHMM* extends the functionality of GPCRHMM by mapping GPCRHMM’s global scores to likelihood scores using a discrete likelihood score function.

GPCRHMM was run on the combined data set sequences to compute global and local scores. Analysis of the global and local scores indicated that the global score is an effective predictor of a given input sequence’s known classification but the correlation did not fit a simple function (Additional file 1: Figure S1).

GPCRHMM’s classification algorithm is as follows: GPCRHMM was run on training set sequences with known classifications (GPCR / non-GPCR). The discrete likelihood score function was computed (trained) using the global scores for the training set sequences. The discrete likelihood score function was represented by partitioning the range of global scores into 100 intervals of equal width. A likelihood score was computed for each interval by dividing the number of known GPCRs in each interval by the total number of sequences with global scores in each interval. During the classification stage, a global score was computed for each sequence using GPCRHMM. The sequences’ global scores were mapped to likelihood scores by identifying the interval with the appropriate range.

The computation of GPCRHMM*’s discrete likelihood score function (*L*_GPCRHMM*_) for a sequence *x* can be expressed using the following formula:

$$\begin{array}{l} L_{\mathrm{GPCRHMM}*}\left(x\;\mathrm{is}\;a\;\mathrm{GPCR}\left|x_{\mathrm{global}\_\mathrm{score}}\right)\right)\\ \;=\frac{\#\;\mathrm{of}\;\mathrm{GPCRs}\;\mathrm{in}\;\mathrm{bin}\;\left(x_{\mathrm{global}\_\mathrm{score}}\right)}{\#\;\mathrm{of}\;\mathrm{all}\;\mathrm{sequences}\;\mathrm{in}\;\mathrm{bin}\;\left(x_{\mathrm{global}\_\mathrm{score}}\right)} \end{array}$$

#### Pfam* classifier

Pfam* maps whole-protein expectation value (e-value) computed using Pfam A GPCR clan HMMs (retrieved from the Pfam database [26] (Additional file 1: Table S3) to likelihood scores using a discrete likelihood score function.

We used HMMER [55] to compute the e-values for the sequences in the combined training set against the Pfam A GPCR clan HMMs. We then analyzed the resulting distribution of e-values with respect to the known classifications for the sequences in the combined training set. In the case of matches against multiple HMMs for a single sequence, we selected the lowest e-value. (Smaller e-values indicate better agreement.) We found that the distribution of the logarithms of the e-values could be used to accurately discriminate between GPCRs and non-GPCRs. (Additional file 1 Section 2 contains a discussion of the analysis of the e-value distributions). We used HMMER’s default threshold for the e-values; any sequences for which e-values were not reported (the e-values were larger than the threshold) were classified as non-GPCRs.

Pfam’s classification algorithm is as follows: During the training stage, the Pfam A GPCR clan HMMs were run on all of the training set sequences. In the case of matches against multiple HMMs, the lowest computed e-value for each sequence is used as the e-value for that sequence. The logarithm of each e-value was then computed. The discrete likelihood score function was represented by partitioning the range of e-value logarithms into 100 intervals of equal width. A likelihood score was computed for each interval as the number of GPCRs divided by the total number of sequences with e-value logarithms in that interval. During the classification stage, the Pfam A GPCR clan HMMs were run against each input sequence, and the lowest computed e-value was taken. The log of the e-value was then mapped to a likelihood score by identifying the interval with the appropriate range.

The computation of Pfam*’s discrete likelihood score function (*L*_Pfam*_) for a sequence *x* can be expressed using the following formula:

$$\begin{array}{l} L_{\mathrm{pfam}*}\left(x\;\mathrm{is}\;\mathrm{aGPCR}\left|x_{\mathrm{evalue}}\right)\right)\\ \;=\frac{\#\;\mathrm{GPCRs}\;\mathrm{in}\;\mathrm{bin}\left(\log\left(x_{\mathrm{evalue}}\right)\right)}{\#\;\mathrm{of}\;\mathrm{all}\;\mathrm{sequences}\;\mathrm{in}\;\mathrm{bin}\left(\log\left(x_{\mathrm{evalue}}\right)\right)} \end{array}$$

#### Ensemble* classifier

Ensemble* computed a discrete likelihood score for each input sequence as a linear combination of the Ensemble* Classifier. Ensemble* computed a discrete likelihood score for each input sequence as a linear combination of the likelihood scores computed by GPCRHMM* and Pfam*.

$$L_{\mathrm{Ensemble}*}\left(x\right)=\alpha L_{\mathrm{Pfam}*}\left(x\right)+\left(1-\alpha \right)L_{\mathrm{GPRCRHMM}*}\left(x\right)$$

The function L_Ensemble*_ (*x*) computes the predicted likelihood score that a given sequence *x* is a GPCR. The functions L_Pfam*_(*x*) and L_GPCRHMM*_(*x*) are the likelihood scores that *x* is a GPCR as predicted by the Pfam* and GPCRHMM* classifiers, respectively. The variable α, where 0 ≤ α ≤ 1, determines the relative weight of the two classifiers in computing the overall likelihood. More complex weighting schemes were not considered, as this simple linear weighting performed well with α = 0.5. (Additional file 1 Section 3 contains an analysis of different values.)

### Prediction and validation pipeline

Potential GPCRs from *Ae. aegypti*, *An. gambiae*, and *Pe. humanus* were initially identified using Ensemble*. Ensemble* was trained on the combined data set. A multi-step validation was performed on the GPCRs predicted by the Ensemble* classifier (Figure 4). First, database annotations were obtained for all positive predictions and ScanPROSITE [63] was used to confirm the presence of a GPCR domain or profile. A likelihood threshold value for the Ensemble* likelihood score was chosen after this step using the Minimum Error Rate method [21] (as determined by ScanPROSITE and database annotation). The threshold value of 0.085 was chosen independently for each vector, despite the differences between the vectors. All sequences that contained domains or profiles other than GPCR domains or profiles, or which were identified as something other than GPCRs in the database were filtered out. For the remaining sequences, two other validations were performed: similarity searches using BLAST against the NCBI nr database [64] and structure prediction using I-TASSER [57,65], a program for predicting 3D atomic structures from amino acid sequences and function through structural matches to proteins for which the structures and functions are known. Lastly, sequences that were predicted as GPCRs by two out of three other criteria (unambiguous BLAST results indicating similarity to a known GPCR, presence of a GPCR domain or profile as identified by ScanPROSITE, or a high I-TASSER TM-score to a known GPCR) were considered to be confirmed GPCRs. Annotations for newly-discovered GPCRs were submitted to VectorBase.

### Expression analysis of predicted GPCR genes

Vector sequences predicted as GPCRs with likelihood values above the threshold and that also had ScanPROSITE predicted GPCR domains or that were annotated as GPCRs in VectorBase [46] were selected for similarity searches against the available Expressed Sequence Tag (EST) datasets in VectorBase using the BLAST search algorithm. *Ae. aegypti* sequences without an EST match were then selected for confirmation of expression by quantitative real-time PCR.

The objective was to assess whether the predicted GPCRs correspond to expressed genes.

Total RNA was isolated from whole female, and female heads of *Ae. aegypti* mosquitoes using the Trizol reagent (Invitrogen). DNAse treated (Fermentas) total RNA was used as a template for first strand synthesis using oligo (dT) and SuperscriptIII (Invitrogen). Real-time PCR was performed using SybrGreen (ABI) with an ABI 7900 Real-Time PCR System. Real-time PCR was carried out using primers (Additional file 1: Table S4) designed to the various GPCRs spanned by introns, where possible, and the internal control gene, 40S Ribosomal Subunit 5. Each GPCR and control was carried out in quadruplicate for both whole bodies and heads. Experimental cycle threshold (C_T_) values were normalized to 40S Ribosomal Subunit 5 C_T_ values.

## Competing interests

The authors declare no conflicting interests.

## Authors’ contributions

RJN developed and evaluated the classifiers, participated in the independent validation, and worked on the manuscript. JLA performed the independent validation and worked on the manuscript. DAS performed the quantitative RT-PCR and expression analysis. BA performed an initial analysis of existing classifiers. MW and GS maintained the *Ae. aegypti* colony used in the quantitative RT-PCR. FHC conceived of the project and participated in the design of the study. MAM and JAI conceived of the project, participated in the design of the study, and worked on the manuscript. All authors have read and approved the final manuscript.

## Supplementary Material

Additional file 1 Supplemental information concerning the Ensemble* classifier. Click here for file
