# Supplementary material for: Identification of novel arthropod vector G protein-coupled receptors
Source: Parasit Vectors. 2013 May 24;6:150. doi: 10.1186/1756-3305-6-150 (PMC3680159; doi:10.1186/1756-3305-6-150)
Supplement: Additional file 1 — Supplemental information concerning the Ensemble* classifier. [file 1756-3305-6-150-S1.pdf]

Supplemental Materials for *Identification of Novel Arthropod Vector GPCRs*

January 23, 2013

1 Supplemental Tables

Table 1: Overall Independent Validation Summary.

	Ae. aegypti	***An. gambiae***	***Pe. humanus***
ScanPROSITE and/or Database confirmation	4	16	7
No ScanPROSITE and no Database annotation	8	9	8
Known GPCRs	2	3	0
Novel Sequences Confirmed by BLAST as GPCRs	2	13	1
Novel sequences likely to be GPCRs by BLAST	2	3	2
Novel sequences unlikely to be GPCRs by BLAST	4	4	8
NOT GPCRs by BLAST	0	1	2
Unable to validate by BLAST	2	1	2
Confirmed by I-TASSER	1	9	5
NOT GPCRs by I-TASSER	5	12	10
Unconfirmed by I-TASSER	0	1	1
Sequences confirmed by all validation methods	1	6	0
Total	12	25	15

Table 2: *Ae. aegypti* EST Expression Analysis.

**Prediction Value**	**Sequence ID**	**Database Annotation**	**ScanPROSITE**	**Expression Head***	**Expression Body***
0.982	AAEL012356-PA	GPCR Somatostatin Family	G-protein coupled receptors family 1	++	+/-
0.943	AAEL005373-PA	GPCR (Rhod)opsin Family	G-protein coupled receptors family 1	++	+/-
0.933	AAEL009673-PA	GPCR Gonadotrophin Releasing Hormone Family	G-protein coupled receptors family 1	++	+/-

Prediction Value	Sequence ID	Database Annotation	ScanPROSITE	Expression Head*	Expression Body*
0.900	AAEL017048-PA	GPCR Growth Hormone Releasing Hormone Family	G-protein coupled receptors family 1	+++	+
0.864	AAEL002894-PA	GPCR Purine/Adenosine Family	G-protein coupled receptors family 1	+++	++
0.859	AAEL012796-PA	GPCR Growth Hormone Releasing Hormone Family	G-protein coupled receptors family 1	++	+
0.857	AAEL008282-PA	GPCR Neurokinin/Tachykinin Family	G-protein coupled receptors family 1	+/-	++
0.852	AAEL007169-PA	Allatostatin receptor	G-protein coupled receptors family 1	+++	+
0.838	AAEL010626-PA	GPCR Neuropeptide Y Family	G-protein coupled receptors family 1	++	+
0.798	AAEL004399-PA	GPCR Glycoprotein Hormone Family	G-protein coupled receptors family 1	+	+
0.769	AAEL003747-PA	GPCR Growth Hormone Releasing Hormone Family	G-protein coupled receptors family 1	++	+
0.736	AAEL008267-PA	GPCR Neurokinin / Tachykinin Family	G-protein coupled receptors family 1	+/-	++
0.722	AAEL012920-PA	GPCR Galanin / Allatostatin Family	G-protein coupled receptors family 1	+++	++
0.702	AAEL017335-PA	GPCR Growth Hormone Releasing Hormone Family	G-protein coupled receptors family 1	++	+

Prediction Value	Sequence ID	Database Annotation	ScanPROSITE	Expression Head*	Expression Body*
0.638	AAEL006283-PA	GPCR Myosuppressin Family	G-protein coupled receptors family 1	++	+++
0.629	AAEL000266-PA	GPCR Orphan/Putative Class A Family	G-protein coupled receptors family 1	+++	++
0.616	AAEL008652-PA	GPCR Vasopressin Family	G-protein coupled receptors family 1	++	++
0.599	AAEL017167-PA	GPCR HE6-like Family	GPS domain; G_PROTEIN_RECEP_F2_4	++	+
0.573	AAEL008322-PA	GPCR Frizzled / Smoothened Family	Frizzled (fz) domain; G-protein coupled receptors family 2	+/-	+
0.559	AAEL004777-PA	Glycoprotein Hormone Family	17 Leucine-rich repeat ; G-protein coupled receptors family 1	+	++
0.549	AAEL009024-PA	GPCR Calcitonin / Diuretic Hormone Family	2 G-protein coupled receptors family 2	+++	+
0.516	AAEL003473-PA	GPCR Gastrin / Bombesin Family	G-protein coupled receptors family 1	+++	+
0.484	AAEL006832-PA	GPCR Frizzled / Smoothened Family	Frizzled (fz) domain; G-protein coupled receptors family 2	++	++
0.465	AAEL005322-PA	Ultraviolet-sensitive opsin	G-protein coupled receptors family 1	+	+
0.455	AAEL006947-PA	GPCR Neurokinin / Tachykinin Family	G-protein coupled receptors family 1	++	+
0.450	AAEL010207-PA	Sulfakinin receptor (dsk-r1)	G-protein coupled receptors family 1	++	+
0.375	AAEL017238-PA	GPCR Gastrin / Cholecystokinin Family	G-protein coupled receptors family 1	++	+

**Prediction Value**	**Sequence ID**	**Database Annotation**	**ScanPROSITE**	**Expression Head***	**Expression Body***
0.227	AAEL004153-PA	Conserved hypothetical protein	G-protein coupled receptors family 1	++	+
0.346	AAEL013430-PA			++	+++
0.250	AAEL002694-PA			+++	++
0.214	AAEL010852-PA			++	++
0.188	AAEL000818-PA	Conserved hypothetical protein	G-protein coupled receptors family 3	+	+
0.175	AAEL004160-PA	Cap2b receptor, putative	G-protein coupled receptors family 1	-	-
0.167	AAEL017259-PA	GPCR Methuselah Family	No hits	+	+++
0.167	AAEL017410-PA	GPCR Gastrin / Bombesin Family	G-protein coupled receptors family 1	++	+
0.133	AAEL017341-PA	GPCR Neurokinin / Tachykinin Family	G-protein coupled receptors family 1	++	+
0.125	AAEL017414-PA	GPCR Neurokinin / Tachykinin Family	G-protein coupled receptors family 1	++	+
0.013	AAEL013422-PA	Odorant receptor 42a, putative	No hits	+	+

*Expression of each gene was determined by the ΔC_T method, where expression was normalized to 40S ribosomal subunit 5. +++ = ΔC_T 0-9; ++ = ΔC_T 9-12; + = ΔC_T 12-17; +/- = $\Delta C_T > 17$

Table 3: List of Pfam HMMs Used in Pfam* and Ensemble* Classifiers.

**Pfam Family HMM Short Name**	**Pfam Family Description**
7TM_7TMR_HD	7TM receptor with intracellular HD hydrolase
7TM_GPCR_Srh	Serpentine type 7TM GPCR chemoreceptor Srh
7TM_GPCR_Srx	Serpentine type 7TM GPCR chemoreceptor Srx
Git3	G protein-coupled glucose receptor regulating Gpa2
TAS2R	Mammalian taste receptor protein
7tm_1	7 transmembrane receptor (rhodopsin family)
7TM_GPCR_Sri	Serpentine type 7TM GPCR chemoreceptor Sri
7TM_GPCR_Srz	Serpentine type 7TM GPCR chemoreceptor Srz

Pfam Family HMM Short Name	Pfam Family Description
Git3_C	G protein-coupled glucose receptor regulating Gpa2 C-term
V1R	Vomeronasal organ pheromone receptor family, V1R
7tm_2	7 transmembrane receptor (Secretin family)
7TM_GPCR_Srj	Serpentine type 7TM GPCR chemoreceptor Srj
7TM_GPCR_Str	Serpentine type 7TM GPCR chemoreceptor Str
GpcrRhopsn4	Rhodopsin-like GPCR transmembrane domain
7TM_GPCR_Sra	Serpentine type 7TM GPCR chemoreceptor Sra
7TM_GPCR_Srsx	Serpentine type 7TM GPCR chemoreceptor Srsx
Bac_rhodopsin	Bacteriorhodopsin-like protein
Lung_7-TM_R<br>hline 7TM_GPCR_Srab	Lung seven transmembrane receptor<br>Serpentine type 7TM GPCR receptor class ab chemoreceptor
7TM_GPCR_Srt	Serpentine type 7TM GPCR chemoreceptor Srt
Dicty_CAR	Slime mold cyclic AMP receptor
Ocular_alb	Ocular albinism type 1 protein
7TM_GPCR_Srb	Serpentine type 7TM GPCR chemoreceptor Srb
7TM_GPCR_Sru	Serpentine type 7TM GPCR chemoreceptor Sru
DUF1182	Protein of unknown function (DUF1182)
7TM_GPCR_Srbc	Serpentine type 7TM GPCR chemoreceptor Srbc
7TM_GPCR_Srv	Serpentine type 7TM GPCR chemoreceptor Srv
DUF621	Protein of unknown function (DUF621)
Sre	*C. elegans* Sre G protein-coupled chemoreceptor
7TM_GPCR_Srd	Serpentine type 7TM GPCR chemoreceptor Srd
7TM_GPCR_Srw	Serpentine type 7TM GPCR chemoreceptor Srw
Frizzled	Frizzled/Smoothened family membrane region
Srg	Srg family chemoreceptor
STE2	Fungal pheromone mating factor STE2 GPCR

Table 4: *Ae. aegypti* Quantitative RT-PCR Primers.

Primer Name	Primer Sequence
AAEL000266 Forward	GCA CTC ACG CAG GCA ACC CTT ATT
AAEL000266 Reverse	GGA ACG ATG AGC AGA CCG CAT AGC
AAEL000818 Forward	AGT GCG TCT TCT TGA TCA GCC TCA
AAEL000818 Reverse	CGG CAA CTT GAA TCA ACA CTG CGA
AAEL002694 Forward	GGC CAA CGT ACA TGT GGA AAT GAA AAG C
AAEL002694 Reverse	AAT CAC GCC ACT GGA GGA ATG ACA
AAEL002894 b Forward	TCG ATC TTC TGC CTG GTC GCT GTT
AAEL002894 b Reverse	CGA ATA GCG GAA GAA ACC CGA TGA TTG
AAEL003473 b Reverse	GCC ATG AGT CCA CCG TGT AGA GAA
AAEL003473 Forward	TTT ATC GTT GGT GTG CTG GGC AAC
AAEL003747 b Reverse	CGG TGA TGA TGC AAG CCA CTT GGT
AAEL003747 Forward	TTC ATC TTC GTC GTC GGC ATT GTG
AAEL004153 Forward	GGC AAC AAC TGA CGA CTT GTG GAT
AAEL004153 Reverse	TGG AAC TGG AAT CCC TCG ACG TTT
AAEL004160 b Reverse	TGG CAA AGA TGA TCG CCA GCA GAG
AAEL004160 Forward	TTC GGG TGA TAT GGC AAC CGA CAT
AAEL004399 Forward	TGT ATG CCA ACG AGC TGC AGG ATT
AAEL004399 Reverse	CAG CTT GGG TGC ATG TGC TAT GTA

Primer Name	Primer Sequence
AAEL004777 Forward	ATC ACG GTC CAG TGC CAG CAA TTC
AAEL004777 Reverse	CGT GGG CAA GAA ACG TAC ACC CAA
AAEL005322 Forward	TGC TGG AGG CAC CAC TCT TCG TTT
AAEL005322 Reverse	CCT TCA GTC GGG TAG TCG AGC GTT
AAEL005373 Forward	TGA TTG CGC TGA TGT GCA AGG ATG
AAEL005373 Reverse	AAA TGG CCG ATG TCA GTG TGA ACG
AAEL006283 b Forward	CAA GGC ATC CTG GGG CTG CTA AGT
AAEL006283 b Reverse	AAG GCA AGC ACG TCC ATC AGG TCT
AAEL006832 Forward	GTT CGC AAG GAC TGT TTC AGC GAT
AAEL006832 Reverse	TGG TCG TAC TCG AGG GTG TAG TTG AT
AAEL006947 Forward	ACT GGC TGG CGA TGA GTA ATT CGA
AAEL006947 Reverse	TCC TAA TGC AAG GAC ACC ACC T
AAEL007169 Forward	GAC CAC AGC AAT TCC AGT CGC CAT
AAEL007169 Reverse	TCC ATA ACC GAG CCA GCA TTC CGA
AAEL008267 Forward	GGG TTT GTT GCT GCT TTG CCC TTA
AAEL008267 Reverse	GAC ACT CAA CGG AAG CCA AAC CAA
AAEL008282 Forward	AGA TTG ACG ATG CGA GGC GTA ACA
AAEL008282 Reverse	ACC AAA GCT CCA ATC AGG ACA TGC
AAEL008322 b Forward	CGC TTG TGG CTA CGA CAC ACA ACT
AAEL008322 b Reverse	AAC CGA TTT GCT CCA ACG CAA GCA
AAEL008652 b Reverse	CCC GCC AGC CAG GAA ATG GTA ATC
AAEL008652 b Forward	TGT ACT AGG GAA CTT GGC TGT GCT
AAEL009024 Forward	TTA CTG CAA CTG GAC ATG GGA CGA
AAEL009024 Reverse	AGT GGA GTT GGT GTA GTT CCT GGT
AAEL009673 Forward	CCG TCG TGA TAA CCC TGT TTC GAT CA
AAEL009673 Reverse	CCG CAA CGG ATA TAT CAC AGC AAA GC
AAEL010207 b Forward	GCG GAT GCG GAC GGT TAC CAA TG
AAEL010207 b Reverse	CCG AAA CGG ACA CGG CTT GAA AGT
AAEL010626 b Reverse	CGG CAT CGT AAC CAG GCA CAG TAG
AAEL010626 Forward	TAC TGA TCA TAT TTG GCG CCA CGG
AAEL010852 Forward	GGA CGA GGA TAC CCC AGC GAA TGT
AAEL010852 Reverse	ACT GGC AGG TTG TCT GGT TTT GGT
AAEL012356 Forward	ATA GCT GTG TGC CAT CCC ATC TCA
AAEL012356 Reverse	TCT CGA TGA ACC GTG TTG GCG TAT
AAEL012796 b Forward	CGA CGG GTC CTG AAA ATG CTC GTT
AAEL012796 b Reverse	ATG GAT CGT TCG GCT GAT GGT CCT
AAEL012920 Forward	GAA CGA ACT CCG ACA CGA CCC AAC
AAEL012920 Reverse	TTG TAC GAC AGG TGG GCT GGG ATT
AAEL013422 Forward	TGC AGA AAT CGA TGG ACC GCT ACT
AAEL013422 Reverse	CAA GAA GCC GAC CAT GAA GCA CAA
AAEL013430 Forward	CTG TGT CGC GTT TGT CAT GGC TCT
AAEL013430 Reverse	CCG AAG CCG AAC AAA GGG AGA AAC
AAEl014224 Forward	GGA TCA TCA TGC GGT GCT TCT GCT
AAEL014224 Reverse	CAT CTG AAG ACT GTA CTG TCC AGT TCG GTT
AAEL017048 b Forward	GCC ATT TCG GAT TTG CTG TTG CTT T
AAEL017048 b Reverse	CTC CAC TGT GAA GGC TGT TAT CGT T
AAEL017167 Forward	GCC CCG ATT GAT TTT GTT GGT GGT

2 Analysis of Classifier Scores to Determine Likelihood Distributions

The distribution of global scores from GPCRHMM and lowest e-value scores from the Pfam HMMs were analyzed to determine the best way for the GPCRHMM* and Pfam* classifiers to compute likelihood scores. The distribution of global and local scores computed by GPCRHMM for the combined data set was analyzed with respect to the known classification of the sequences. The global score was determined to be the most effective predictor of a given input sequences known classification but the correlation did not fit a simple function (Supplemental Figure 1). Thus, GPCRHMM* uses a discrete likelihood score function to compute likelihood scores from the global scores.

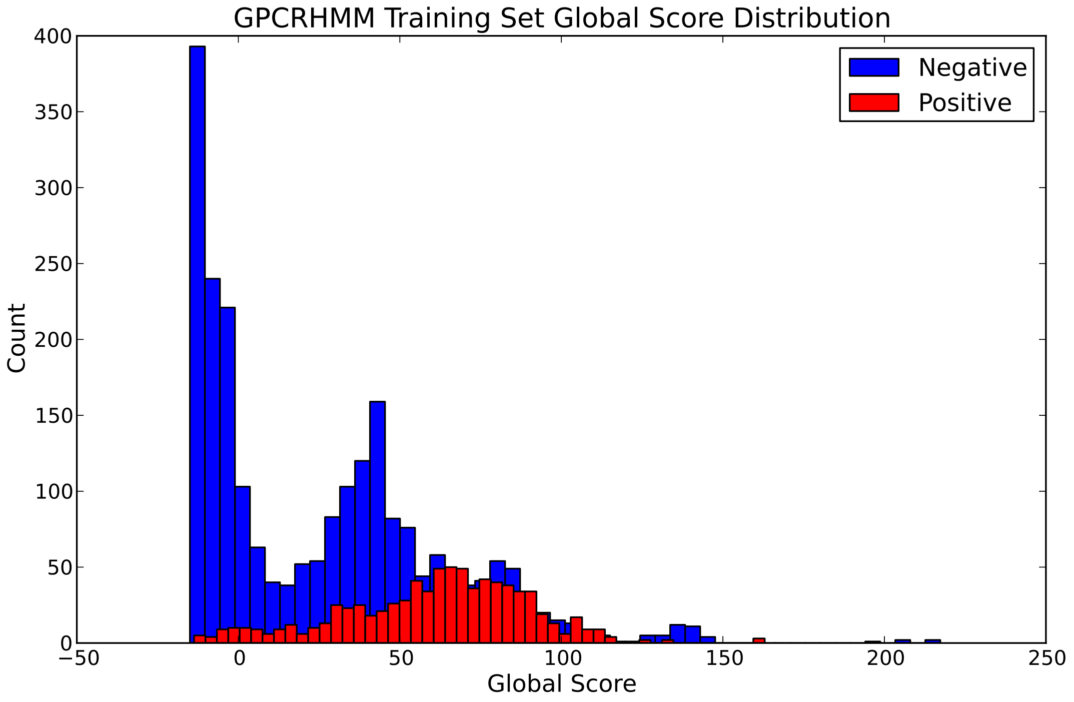

Figure 1: Histogram of GPCRHMM Global Scores Colored by Sequence Type (GPCR – red, not GPCR – blue) for All Organisms

Each Pfam GPCR Clan A HMM (Supplemental Table 3) was run on each training-set sequence, and the lowest e-value (best match) for each training set sequence was selected. The range of Pfam* e-values was too small to directly fit a discrete likelihood score function. However, the distribution of the logarithms of the e-values provided greater separation between the GPCRs and other proteins (Supplemental Figure 2). Thus, the Pfam* uses a discrete likelihood score function applied to the logarithm of the e-values to compute the likelihood scores from the Pfam HMM e-values.

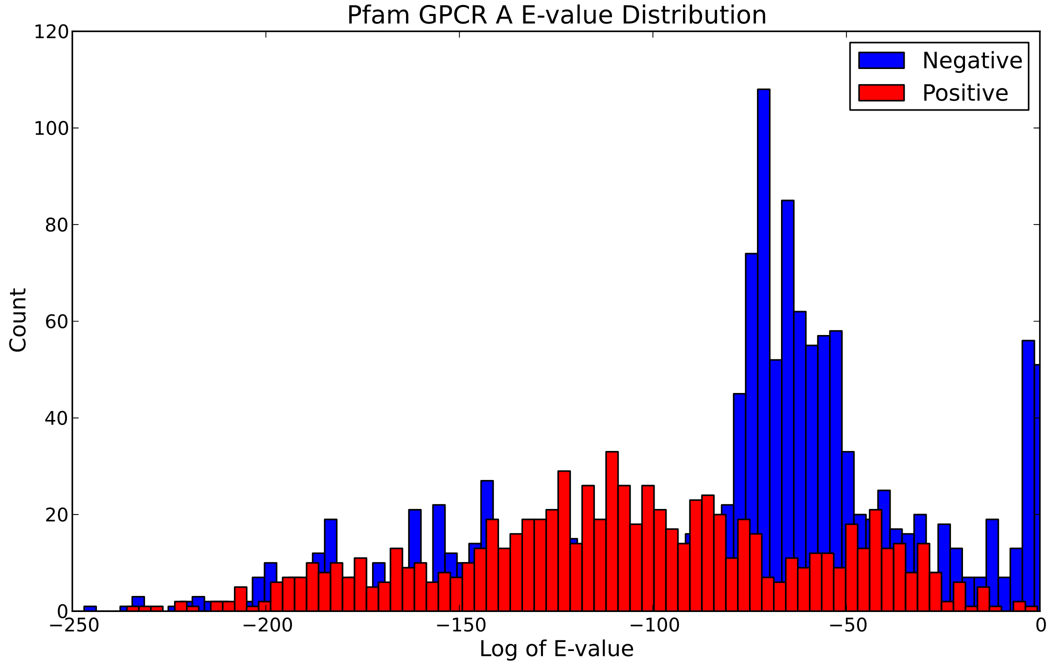

Figure 2: Histogram of log of Pfam GPCR Clan A HMM E-values Colored by Sequence Type (GPCR red, other blue) for All Organisms

3 Likelihood Value from the Ensemble* Classifier Allows Fine-Tuning of Use for Different Applications

Ensemble* was designed to be easily customized for different situations by taking advantage of its parameterized nature. There are three main methods to customize Ensemble* by altering: 1) the composition of the training set; 2) α, a value between 0 and 1 that determines the relative weighting of GPCRHMM* and Pfam* with a higher value favoring Pfam*; 3) and the threshold for the likelihood value for which sequences are accepted as predicted GPCRs. The threshold value represents the likelihood cut off value below which sequences are no longer predicted as a GPCR. Raising the threshold value leads to fewer predicted sequences and at the same time fewer false positive predictions, while lowering the threshold value leads to an overall higher number of putative GPCRs being predicted and a higher false positive rate.

We evaluated the performance of Ensemble* with two *Ho. sapiens* training sets. The first training set, published by Zhang et al. ([1]), consisted of a smaller number of sequences for which the confidence in the GPCR annotations was higher and from which we removed olfactory receptors; this set is referred to as HomoTest1. The second *Ho. sapiens* training set (HomoSet2) consisted of a larger number of sequences including HomoSet1 plus other *Ho. sapiens* sequences annotated as GPCRs in the Ensembl database, but for which we had less confidence in the annotations.

An analysis of the effect of the annotation quality in the training sets on prediction quality was performed. Pfam*'s predictions were more accurate (but had lower sensitivity) with the HomoSet1 and less accurate (but had higher sensitivity) with the HomoSet2, while very little variance was observed in the predictions made by GPCRHMM*. We discovered that running Ensemble* favoring Pfam* ($\alpha = 0.9$) maximized the sensitivity of the predictions when trained with the HomoSet1 (Supplemental Figure 3(a)), while favoring both GPCRHMM* and Pfam* equally ($\alpha = 0.5$) was better for HomoSet2 (Supplemental Figure 3(b)). Despite the reduction in accuracy of Pfam*'s predictions, Ensemble* was able to identify the most GPCRs

when trained with the HomoSet2 and run with $\alpha = 0.5$ (Supplemental Figure 4).

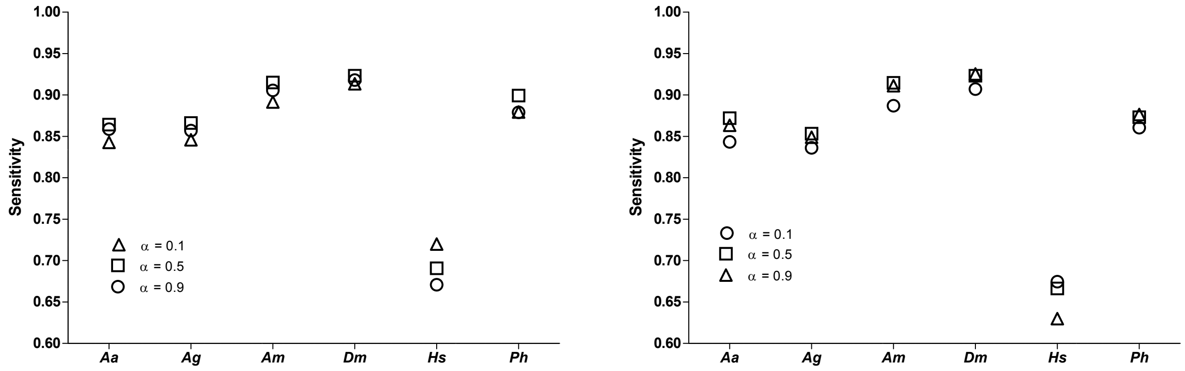

(a) HomoSet1

(b) HomoSet2

Figure 3: Sensitivities (percent of sequences in test set identified as GPCRs), broken down by organism (*Aa* = *Ae. aegypti*, *Ag* = *An. gambiae*, *Ap* = *Ap. mellifera*, *Dm* = *Dr. melanogaster*, *Hs* = *Ho. sapiens*, and *Ph* = *Pe. humanus*), of the Ensemble* classifier when trained with the combined test set using the HomoSet1 (Supplemental Figure 3(a)) and HomoSet2 (Supplemental Figure 3(b)) training sets for *Ho. sapiens* for 2 different α values (0.5, 0.9). The overall difference in the number of identified test set GPCRs is small (1 or 2 sequences) between the HomoSet2 / $\alpha = 0.5$, HomoSet1 / $\alpha = 0.5$, and HomoSet2 / $\alpha = 0.9$. However, Ensemble* is able to identify the most sequences for the insects on average when trained with the HomoSet1 or HomoSet2 test sets and run with $\alpha = 0.5$.

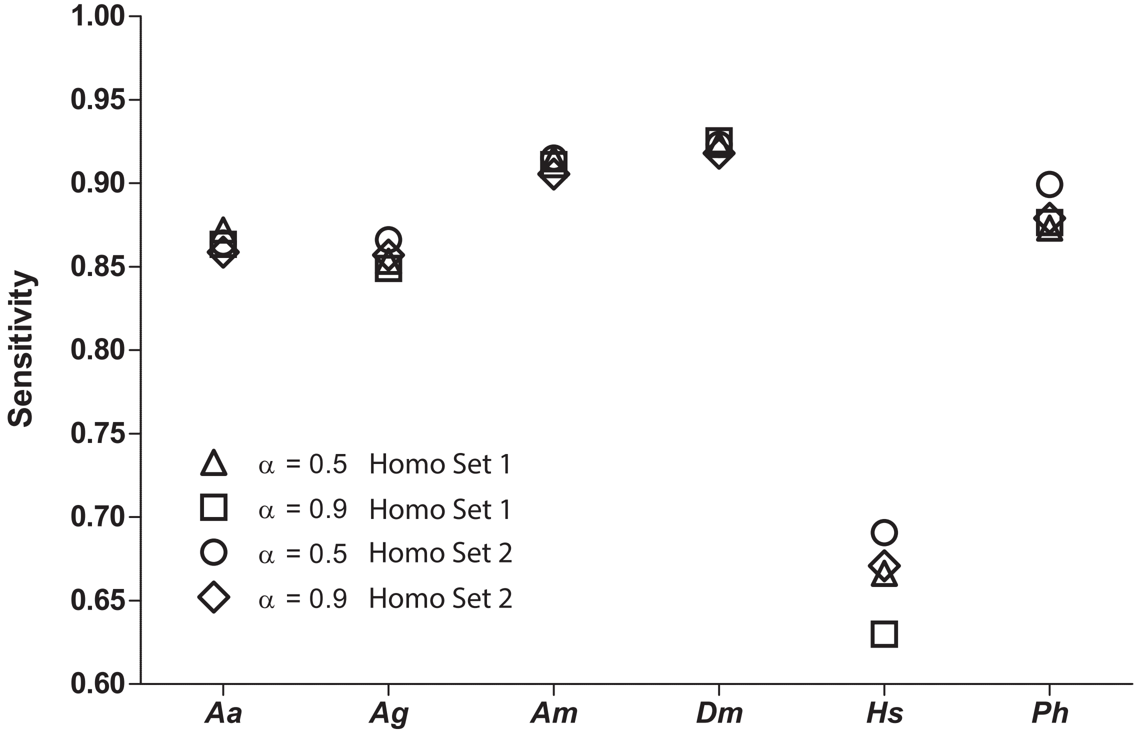

Figure 4: Sensitivities (percent of sequences in test set identified as GPCRs), broken down by organism (*Aa* = *Ae. aegypti*, *Ag* = *An. gambiae*, *Ap* = *Ap. mellifera*, *Dm* = *Dr. melanogaster*, *Hs* = *Ho. sapiens*, and *Ph* = *Pe. humanus*), of the Ensemble* classifier when trained with the combined test set using the HomoSet1 and HomoSet2 training sets for *Ho. sapiens* for 2 different α values (0.5, 0.9). The overall difference in the number of identified test set GPCRs is small (1 or 2 sequences) between the HomoSet1 / $\alpha = 0.5$, HomoSet2 / $\alpha = 0.5$, and HomoSet2 / $\alpha = 0.9$. However, Ensemble* is able to identify the most sequences for the insects on average when trained with the HomoSet1 or HomoSet2 test sets and run with $\alpha = 0.5$.

References

[1] Yang Zhang et al. Structure modeling of all identified G protein-coupled receptors in the human genome. *PLoS computational biology*, 2(2):e13, February 2006.
